# Comparison of the Diagnostic Efficiency of Radial- and Convex-Arrayed Echoendoscopes for Indirect Findings of Pancreatic Cancer: A Retrospective Comparative Study Using Propensity Score Method

**DOI:** 10.3390/cancers13061217

**Published:** 2021-03-11

**Authors:** Yuki Ishikawa-Kakiya, Hirotsugu Maruyama, Kei Yamamoto, Masafumi Yamamura, Kojiro Tanoue, Akira Higashimori, Masaki Ominami, Yuji Nadatani, Shusei Fukunaga, Koji Otani, Shuhei Hosomi, Fumio Tanaka, Noriko Kamata, Yasuaki Nagami, Koichi Taira, Masatsugu Shiba, Toshio Watanabe, Yasuhiro Fujiwara

**Affiliations:** 1 Department of Gastroenterology, Osaka City University Graduate School of Medicine, Osaka 545-8585, Japan; m2077735@med.osaka-cu.ac.jp (Y.I.-K.); yamamoto.kei0517@gmail.com (K.Y.); m2069840@med.osaka-cu.ac.jp (M.Y.); m2063374@med.osaka-cu.ac.jp (K.T.); higamo@med.osaka-cu.ac.jp (A.H.); ominami@med.osaka-cu.ac.jp (M.O.); dada@med.osaka-cu.ac.jp (Y.N.); m1156849@med.osaka-cu.ac.jp (S.F.); kojiotani@med.osaka-cu.ac.jp (K.O.); hosomi.shuhei@med.osaka-cu.ac.jp (S.H.); m2079981@med.osaka-cu.ac.jp (F.T.); m1266151@med.osaka-cu.ac.jp (N.K.); m1151493@med.osaka-cu.ac.jp (Y.N.); koichit@med.osaka-cu.ac.jp (K.T.); watanabet@med.osaka-cu.ac.jp (T.W.); yasu@med.osaka-cu.ac.jp (Y.F.); 2 Department of Medical Statistics, Osaka City University Graduate School of Medicine, Osaka 545-8585, Japan; shiba@med.osaka-cu.ac.jp

**Keywords:** endoscopic ultrasonography, pancreatic cancer, indirect findings, inverse probability of treatment weighting analysis

## Abstract

**Simple Summary:**

Pancreatic cancer (PC) has a poor prognosis; however, diagnosing PC at an earlier stage could improve long-term patient outcomes. Endoscopic ultrasonography (EUS) plays an important role in PC detection, and the indirect findings (caliber change, retention cysts, and dilatation of the branch duct) that are detected by EUS are especially important for the early detection of PC. The aim of this retrospective study was to compare the diagnostic efficacy of radial- and convex-arrayed echoendoscope for the detection rate of indirect findings. As a result, the radial-arrayed echoendoscope was found to be an independent detection factor of indirect findings by multivariate analysis. The radial-arrayed echoendoscope is useful for the detection of indirect findings.

**Abstract:**

Endoscopic ultrasonography (EUS) is useful for detecting early-stage pancreatic cancer. Because the detection of small lesions is difficult, it is important to detect indirect findings, namely caliber change, retention cysts, and dilatation of the branch duct, during the procedure. Although two types of EUS endoscopes are frequently used, there is no comparative study on their efficacy for detecting indirect findings. Therefore, we aimed to compare the diagnostic efficacy of these two types for indirect findings. We retrospectively analyzed 316 consecutive patients who had undergone EUS for pancreaticobiliary disease at a single center between January 2017 and December 2018. The main outcome was the detection rate of indirect findings and its comparison between the two echoendoscope types. This outcome was achieved using the inverse probability of treatment weighting (IPTW) analysis. The detection rate of indirect findings was higher for the radial-arrayed endoscope than for the convex-arrayed echoendoscope (9.2% vs. 2.3% (*p* = 0.02)). The univariate analysis also revealed that the radial-arrayed echoendoscope was significantly superior to the convex-arrayed echoendoscope in terms of the detection of indirect findings (odds ratio, 5.94; 95% confidence interval, 1.68–21.10; *p* = 0.01) after IPTW. After adjustment for magnetic resonance imaging (MRI) and computed tomography (CT), radial-arrayed echoendoscope remained an independent factor for indirect finding detection (odds ratio, 6.04; 95% confidence interval, 1.74–21.00; *p* = 0.01). Finally, five patients who had indirect EUS findings were diagnosed with pancreatic cancer. Our results indicate that the radial-arrayed echoendoscope is useful for the detection of indirect findings.

## 1. Introduction

Pancreatic cancer (PC) has a poor prognosis because of its diagnostic difficulty and rapid progression [1,2]. According to the Union for International Cancer Control, the 5 year survival rate of PC is low, even in stage IA (68.7%), and is better if the PC can be diagnosed at a size <10 mm (80.4%) [3]. Therefore, diagnosing PC at an earlier stage than stage IA could improve long-term patient outcomes. 

Several imaging modalities including abdominal ultrasonography, computed tomography (CT), magnetic resonance imaging (MRI), endoscopic ultrasonography (EUS), and endoscopic retrograde cholangiopancreatography (ERCP) are used to detect PC. Advances in PC detection have increased with advances in imaging examinations; however, the detection of small-sized PCs is challenging. Among imaging examinations, EUS is important in detecting PC [4,5,6]. The detection rate of PC (tumor size < 10 mm) was higher for EUS than for ultrasonography and contrasted-enhanced CT [7]. 

Two types of EUS are frequently used: radial-arrayed and convex-arrayed echoendoscopes [8,9,10]. These scopes have specific characteristics: the radial-arrayed echoendoscope can perform a 360 degree scan, which facilitates the identification of the surrounding organs and vessels. It provides a longitudinal image of the pancreas and main pancreatic duct. In contrast, the convex-arrayed echoendoscope performs a 180 degree scan and a scan along the vertical axis of the pancreas. For the collection of tissue samples, oblique- and forward-viewing echoendoscopes are used. Oblique-viewing echoendoscopes are generally used; however, forward-viewing echoendoscopes are useful in some cases, such as after upper gastrointestinal surgery [11] or for the evaluation of colorectal neoplasms [12]. However, it is difficult to align it with the organ axis [10,13,14]. Few studies have compared the capabilities of the two types of echoendoscopes for detecting indirect findings.

Recently, indirect findings of early PC including caliber change, retention cysts, and dilatation of the branch duct were detected on EUS [15]. Furthermore, a slightly low echoic lesion caused by localized pancreatitis and fibrosis has been detected around the PC in situ (PCIS) [16,17,18]. These may play an important role in the detection of indirect findings for early PCs of less than 10 mm. These studies used a radial-arrayed echoendoscope; however, the usefulness of radial-arrayed echoendoscopes for the detection of indirect findings is unknown. Therefore, we hypothesized that the radial-arrayed echoendoscope is more performant than the convex-arrayed echoendoscope in detecting indirect findings. We aimed to compare the diagnostic efficacy of radial-arrayed echoendoscope and convex-arrayed echoendoscope in terms of the detection rate of indirect findings.

## 2. Materials and Methods

### 2.1. Patients and Study Design

This retrospective, single-center, comparative study included patients who underwent EUS for further examination of pancreaticobiliary disease at the Department of Gastroenterology, Osaka City University Hospital, between January 2017 and December 2018. EUS was performed to either confirm pancreaticobiliary disease suspected by another modality (CT, MRI, US, and so on) or during a follow-up examination. We excluded patients who had received post-gastrointestinal surgery, because these cases are different from normal situations, and the EUS images are difficult to evaluate due to the effect of the surgery. We also excluded patients in whom EUS was aborted due to sedation failure and adverse events.

### 2.2. Main Outcome Measurements

We compared the detection rate of indirect findings between the radial-arrayed echoendoscope and convex-arrayed echoendoscope. 

### 2.3. Data Collection

We checked the electronic medical records of the patients for their age, sex, symptoms, drinking habit, smoking habit, history of diabetes mellitus, MRI test within six months, CT test within six months, part of the main lesion (such as tumor and/or the cyst, which must be observed carefully), type of scope, early chronic pancreatitis, chronic pancreatitis, indirect findings (caliber change, retention cysts, dilatation of the branch duct with slightly low echoic lesion), part of the indirect findings, endoscopists, and final diagnosis. 

### 2.4. Definition of Indirect Findings

**Figure 1:**
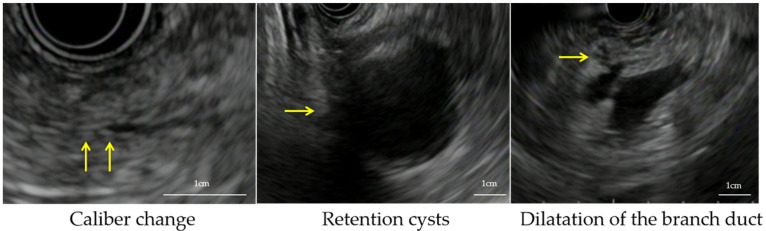
The definition of indirect findings: caliber change, retention cysts, and dilatation of the branch duct.

We defined the indirect findings as follows: caliber change, retention cyst, and dilatation of the branch duct with a slightly low echoic lesion (Figure 1) [16,17,18]. 

We did not include typical pancreatic cancer findings and clear space-occupying lesions that could be diagnosed by EUS as indirect findings.

### 2.5. Endoscopic Procedure and Endoscopists

**Figure 2:**
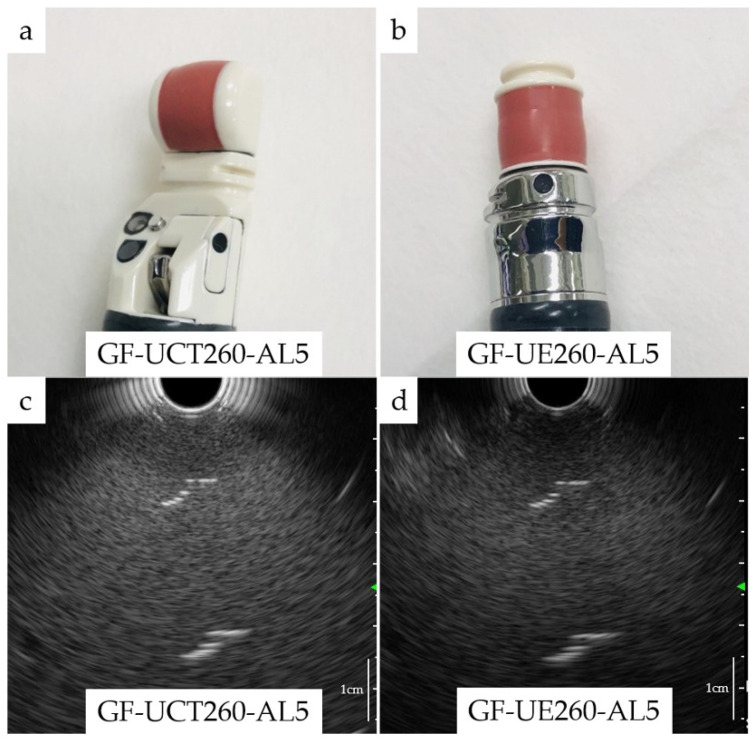
Two types of echoendoscopes and the image of a thread. (**a**) Convex-arrayed array echoendoscope (GF-UCT240-AL5/GF-UCT260-AL5, Olympus Corporation, Tokyo, Japan); (**b**) radial-arrayed echoendoscope (GF-UE260-AL5, Olympus Corporation, Tokyo, Japan); (**c**) image of a thread hanging in water by GF-UCT260-AL5; (**d**) image of a thread hanging in water by GF-UE260-AL5.

All patients underwent EUS. The echoendoscopes (radial-arrayed or convex-arrayed) were chosen by endoscopists. They chose the echoendoscope according to their skills and experience. When a EUS-fine-needle aspiration (FNA) was needed, it was performed during a separate session. Patients were administered an intravenous injection of midazolam (2–10 mg) and/or flunitrazepam (0–2 mg) and pentazocine (0–15 mg), depending on their age and tolerance levels. The procedures were carried out with a convex-arrayed echoendoscope (GF-UCT240-AL5/GF-UCT260-AL5, Olympus Corporation, Tokyo, Japan) (Figure 2a), a radial-arrayed echoendoscope (GF-UE260-AL5, Olympus Corporation, Tokyo, Japan) (Figure 2b) connected to a ProSound F75 (Hitachi, Ltd, Tokyo, Japan), an EU-ME1 (Olympus Corporation, Tokyo, Japan), or an EU-ME2 PREMIER PLUS (Olympus Corporation, Tokyo, Japan).

We defined the endoscopists who met all of the following essential criteria as supervisors: ≥8 years of endoscopy experience, ≥100 EUS examinationyear, and ≥500 radial-arrayed echoendoscope examinations or ≥250 convex-arrayed echoendoscope examinations. The other endoscopists were classified as trainees. The trainees had performed EUS examinations for at least 2 years and performed ≥50 EUS examinations per year [10]. At least one supervisor checked the examination in real-time and changed the scope if necessary.

### 2.6. Resolution of Endoscopes

According to the package insert, the resolution of the two echoendoscopes was the same when connected to the UE-ME1: an axial resolution of <1 mm and lateral resolution of <3 mm by incorporating relatively low-frequency transmission (5–7.5 MHz). Furthermore, there was no difference between the two scopes in terms of visibility as we observed the same thread in water using GF-UCT260 and GF-UE260-AL5 (Figure 2c,d).

### 2.7. Image Evaluation

We confirmed the inter-observer and intra-observer agreement of the indirect findings. We randomly selected 30 cases (10 patients with indirect findings and 20 patients without indirect findings). Two endoscopists (Y.I.-K. and H.M.) assessed the presence of indirect findings as an inter-observer by checking the images. Then, one endoscopist (Y.I.-K.) assessed the same images again as an intra-observer.

### 2.8. Statistical Analysis

Continuous variables are presented as medians and were analyzed using the Fisher’s test, while categorical variables are presented as numbers and were analyzed using the χ^2^ test. The model included age, sex, symptoms, drinking habit, smoking habit, history of diabetes, MRI test in six months, CT test in six months, part of the main lesion, type of scope, early chronic pancreatitis, chronic pancreatitis, indirect findings, and endoscopists. For each factor, we calculated the odds ratio (OR) with a 95% confidence interval (CI). There were no definite factors associated with the detection rate of indirect findings. Previous imaging tests provided information such as caliber changes, retention cysts, and dilatation of the branch duct. Since the CT and MRI might cause information bias in the detection rate of indirect findings, we adjusted them using multivariate analysis.

Kappa coefficients were calculated to assess the inter- and intra-observer agreement of indirect findings. A κ-value of less than 0.50 was regarded as poor agreement, between 0.5 and 0.75 as moderate, between 0.75 and 0.90 as good, and more than 0.90 as excellent agreement [19].

Further, we created a pseudo-population using the inverse probability of treatment weighting (IPTW) method, which is based on propensity scores, and reduced selection bias without reducing the sample size. The IPTW was calculated as the inverse of the conditional probability [20,21]. We evaluated the reliability of the model by using the Hosmer–Lemeshow test for goodness-of-fit statistical analysis.

All statistical analyses were performed using SPSS^TM^ software (Version 26.0, SPSS Inc., Tokyo, Japan) for Windows and the R^TM^ statistical package. All statistical tests were two-sided, and differences were considered statistically significant when *p-*values were <0.05.

## 3. Results

### 3.1. Baseline Characteristics of Patients

**Figure 3:**
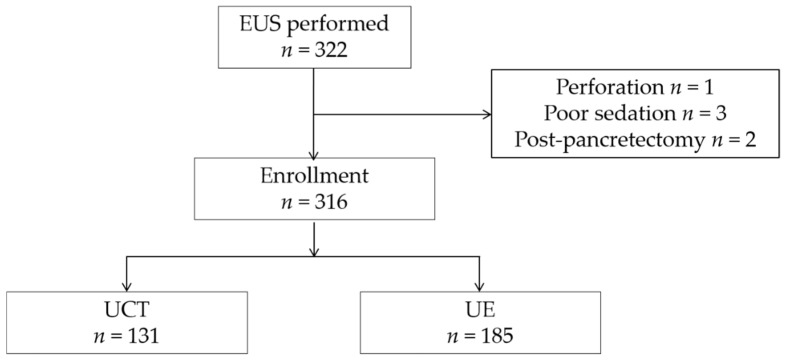
Flow diagram of the enrolled patients. EUS, endoscopic ultrasonography.

We enrolled 322 patients who underwent EUS. Six patients were excluded because of a poor study (three underwent poor sedation, two were post-pancreatectomy, and one had a perforation). We finally included 316 patients (Figure 3). Table 1 shows the clinical characteristics of the patients. The patients were divided into two groups: 185 patients in the radial-arrayed echoendoscope group and 131 patients in the convex-arrayed echoendoscope group. Twenty-five patients with typical pancreatic cancer and eight with intraductal papillary mucinous carcinoma (IPMC), who did not have indirect findings, were included in this model. Almost all patients underwent CT and/or MRI examinations in the six months prior to enrollment; however, images were unavailable for 27 patients (8.5%). Some of these 27 patients were referred from another hospital, and we were unable to refer to their images because they did not bring a compact disk recordable with the images to our hospital.

### 3.2. Main Outcome Measurements

#### 3.2.1. The Detection Rate for Indirect Findings

We found indirect findings in 17 patients (9.2%) using the radial-arrayed echoendoscope, and in 3 patients (2.3%) using the convex-arrayed echoendoscope. The detection rate of indirect findings was significantly higher using the radial-arrayed echoendoscope than when using the convex-arrayed echoendoscope (*p* = 0.02). Second, we compared the efficacy of the two echoendoscopes in detecting indirect findings. Regarding each indirect finding, the radial-arrayed echoendoscope detected caliber changes and slightly low echoic lesions better than the convex-arrayed echoendoscope (*p* = 0.01, *p* = 0.02; Table 2).

On univariate analyses, the radial-arrayed echoendoscope significantly detected indirect findings compared with the convex-arrayed echoendoscope (OR, 4.32; 95% CI, 1.24–15.10; *p* = 0.02; Table 3). After IPTW, the radial-arrayed echoendoscope was an independent factor in diagnosing indirect findings (OR, 5.94; 95% CI, 1.68–21.10; *p* = 0.01). Upon CT and MRI adjustment, the radial-arrayed echoendoscope was also an independent detection factor of indirect findings by multivariate analysis (OR, 6.04; 95% CI, 1.74–21.00; *p* = 0.01; Table 4). We evaluated the inter-observer agreements in detecting indirect findings between two endoscopists (Y.I.-K. and H.M.) and found a good inter-observer agreement (κ-value = 0.79).

#### 3.2.2. Final Diagnosis

We further examined 16 patients with indirect findings (single cytology by endoscopic retrograde cholangiopancreatography (ERCP) in four, serial pancreatic juice aspiration cytology examination (SPACE) by ERCP in nine, EUS-fine-needle aspiration biopsy in two, surgery in one). We found that four patients with PC were staged pT1b, pT1c, pT2, and pT3, respectively, and one had IPMC staged pTis (Table 5).

#### 3.2.3. Evaluation Using IPTW

We created a quasi-randomized study using IPTW. Subjects were randomly assigned to each group so that the background characteristics were likely similar between the radial-arrayed and convex-arrayed groups. The Hosmer–Lemeshow test revealed that the propensity-weighted model was well-calibrated (*p* = 0.32).

## 4. Discussion

We investigated the hypothesis that a radial-arrayed echoendoscope would be superior in detecting indirect findings to a convex-arrayed echoendoscope. We found that the detection rate of the radial-arrayed echoendoscope for indirect findings was superior to that of the convex-arrayed echoendoscope. In particular, the radial-arrayed echoendoscope had a significantly higher detection rate for caliber changes and slightly low echoic lesions. To the best of our knowledge, this is the first report focusing on the efficacy of the radial-arrayed and convex-arrayed echoendoscopes in detecting indirect findings using IPTW.

EUS is known to have a high ability to detect PC [4,5,6,7]. The Clinical Practice Guidelines for Pancreatic Cancer 2019 from the Japan Pancreas Society recommends EUS to patients with suspected PC [22]. However, the capabilities of the two types of echoendoscopes were not defined clearly. It was reported that the convex-arrayed echoendoscope is useful for detecting pancreatic diseases [23]. Conversely, the ability of both echoendoscopes to stage pancreatic cancer is reportedly equivalent [24]. In the West and Japan, convex-arrayed echoendoscopes are usually used because of the aspect of tissue correction [25]. However, their capability to detect indirect findings has not been investigated, and additional knowledge about the characteristics of these echoendoscopes is required to select an appropriate echoendoscope for the patient. To detect early PC or PCIS, it is necessary to find slight changes in the pancreatic duct such as caliber change, retention cyst, and dilatation of the branch duct by EUS. In addition, inflammation is caused by localized pancreatitis and fibrosis around the PCIS, and these were found to be slightly low echoic lesions [16,18]. These indirect findings are important factors in detecting early PC or PCIS. Concerning the detection of indirect findings, there was no clear adaptation for the selection of echoendoscopes. In the present study, a radial-arrayed echoendoscope was found to be significantly better than a convex-arrayed echoendoscope. For this reason, we assumed that the ability of the radial-arrayed echoendoscope to visualize long-axis images makes detection of these indirect findings possible. Slight changes in the main pancreatic duct, such as caliber changes and dilatation of the branch duct, were visualized easily by long-axis imaging. When we find these changes, we need to check this part in the magnified image and identify a hypoechoic lesion. In addition, we found many indirect findings in areas other than the main lesion using radial-arrayed echoendoscopes. This supports the finding that the radial-arrayed echoendoscope is suitable for detecting indirect findings. In contrast, a convex-arrayed echoendoscope visualizes a short-axis image of the pancreas. Therefore, since the convex-arrayed type cannot visualize the pancreatic duct at a long distance, it may be difficult to visualize the continuous slight changes in the pancreatic duct. In this study, we indicated the usefulness of the radial-arrayed echoendoscope; however, our findings may not be consistent with those of existing reports [23]. This may be because we focused on slight changes rather than clear lesions.

In addition to EUS, MRI is a useful modality to detect slight changes in the pancreatic duct. It is a non-invasive examination and can detect caliber change and dilatation of the branch duct [16,26]. However, it is not useful for visualizing small inflammation, fibrosis, and extremely small PCs. In our study, six cases showed no indirect findings could not be detected any indirect findings by CT and/or MRI. Furthermore, slightly low echoic lesions could not be detected by MRI and/or CT and were instead detected by radial-arrayed echoendoscopes. Slightly low echoic lesions can be caused by small PCs, inflammation, or fibrosis caused by PCIS. Therefore, there is a need to detect these indirect findings and further examine them to identify early PC. As an additional diagnostic examination, EUS-fine-needle aspiration biopsy (EUS-FNAB) is useful. Even though the PC was small (<10 mm), the diagnostic yield was reported to be 96% [27]. If EUS-FNAB is not useful, the lesion might be inflammation or fibrosis. In this case, an alternative method is needed, such as SPACE, which has been reported to be effective for the early diagnosis of PC [28,29]. We performed SPACE in nine patients and found two cases of PC in this study.

We found four patients with invasive ductal carcinoma (IDC) and one patient with IPMC. In the present study, indirect findings could aid in diagnosing early PC. All PC cases had caliber changes and slightly low echoic lesions. These indirect findings could be useful for detecting PC. 

This study has three strengths: First, we found that radial-arrayed echoendoscope was an appropriate modality to detect indirect findings. No prior reports examined the association between radial-arrayed and convex-arrayed echoendoscopes while focusing on indirect findings. Second, we attempted a quasi-randomization using the IPTW method by the propensity score to minimize bias from confounding variables [30]. Third, many hospitals do not have an expensive echoendoscope of each type. Our institution has both echoendoscopes and receives about 500 cases/year. Therefore, we have sufficient experience in pancreatic disease evaluation. 

There were several limitations to this study: First, there were only 20 patients with indirect findings that cannot point out cancer itself. However, according to the Onomichi project, the detection rate of early pancreatic cancer with an indirect finding is only 0.008% [16]. Therefore, it is challenging to correct patients with indirect findings. Further research using a multi-center study is needed. Second, propensity score analysis is a statistical method of adjusting data for selection bias in observational studies and approximates randomized trial approaches. It has an inherent limitation of the choice of a finite number of covariates, which may lead to the omission of relevant covariates. We think that the most likely confounders were identified in our study, although we recognize that it is difficult to adjust for potential confounders using propensity score analysis. Third, this study included some patients who underwent EUS using an UCT240. There were no detailed data about the resolution of the UCT240, but it may have a lower resolution than the UE260 endoscope. This would affect the detection rate of indirect findings. Fourth, even though EUS was performed under expert supervision, bias may exist in the diagnostic agreement on indirect findings, chronic pancreatitis, and early chronic pancreatitis. The κ-test was performed to investigate the reproducibility of only indirect findings, and a good inter-observed agreement was noted; however, other factors such as chronic pancreatitis and early chronic pancreatitis may have influenced the detection of indirect findings.

## 5. Conclusions

In conclusion, the radial-arrayed echoendoscope is useful for detecting indirect findings of PC at an early stage. It might be better to use a radial-arrayed echoendoscope for observation of the pancreas without the need for sampling PCs.

## Figures and Tables

**Table 1:** The clinical characteristics of the patients.

Characteristics	Category	Radial-Type *n* = 185, (%)	Convex-Type *n* = 131, (%)	*p* Value
Age (median)		71	70	0.60
Sex	male	83 (44.9)	64 (48.9)	0.49
Symptom	yes	20 (10.8)	22 (16.8)	0.13
Smoking habit	yes	36 (19.5)	34 (26.0)	0.22
Drinking habit	yes	43 (23.2)	39 (29.8)	0.20
DM	yes	42 (22.7)	25 (19.1)	0.49
Previous CT	yes	112 (60.5)	91 (69.5)	0.12
Previous MRI	yes	109 (58.9)	80 (61.1)	0.73
Part of the main lesion				0.16
	all	6	0	
	uncinate process	12	15	
	head	57	37	
	neck	20	15	
	body	51	42	
	tail	39	22	
Early chronic pancreatitis	yes	30 (16.2)	14 (10.1)	0.19
Chronic pancreatitis	yes	16 (8.6)	5 (3.8)	0.11
Scopist	expert	8 (4.3)	9 (6.9)	0.33

DM: diabetes mellitus, CT: computed tomography, MRI: magnetic resonance imaging.

**Table 2:** Indirect findings between radial-arrayed and convex-arrayed echoendoscope.

Indirect Findings	Radial-Type *n* = 185, (%)	Convex-Type *n* = 131, (%)	*p* Value
Patients based indirect findings			
Indirect findings	17 (9.2)	3 (2.3)	0.02
Lesions based indirect findings			
Caliber change	15 (8.1)	2 (1.5)	0.01
Branch duct dilatation	8 (4.3)	1 (0.8)	0.09
Retention cyst	1 (0.5)	0	1.00
Slightly low echoic lesion	14 (7.6)	2 (1.5)	0.02

**Table 3:** Univariate and multivariate conditional logistic regression analysis of indirect findings.

Variable	Univariate Analysis		Multivariate Analysis	
	Crude OR (95% CI)	*p* Value	Crude OR (95% CI)	*p* Value
Age	1.00 (0.97–1.05)	0.73		
Sex (male)	1.79 (0.71–4.50)	0.22		
Smoking habit	0.87 (0.28–2.70)	0.81		
Drinking habit	0.87 (0.23–2.15)	0.53		
DM	1.26 (0.44–3.60)	0.67		
Symptom	1.16 (0.33–4.15)	0.82		
Previous CT	0.83 (0.33–2.08)	0.68	1.13 (0.43–2.97)	0.81
Previous MRI	2.10 (0.75–5.94)	0.16	2.26 (0.77–6.64)	0.14
Early chronic pancreatitis	0.67 (0.15–3.00)	0.60		
Chronic pancreatitis	1.62 (0.35–7.54)	0.54		
Scope (radial)	4.32 (1.24–15.10)	0.02	4.48 (1.27–15.70)	0.02
Scopist (expert)	0.92 (0.12–7.31)	0.94		

DM: diabetes mellitus, CT: computed tomography, MRI: magnetic resonance imaging, OR: odds ratio, CI: confidence interval.

**Table 4:** Univariate and multivariate conditional logistic regression analysis of indirect findings after IPTW.

Variable	Odds Ratio (95% CI)	*p* Value
Before IPTW		
Unadjusted (radial/convex)	4.32 (1.24–15.10)	0.02
Adjusted for previous CT, previous MRI	4.48 (1.27–15.70)	0.02
After IPTW		
Unadjusted (radial/convex)	5.94 (1.68–21.10)	0.01
Adjusted for previous CT, previous MRI	6.04 (1.74–21.00)	0.01

IPTW: inverse probability of treatment weighting, CT: computed tomography, MRI: magnetic resonance imaging.

**Table 5:** Twenty cases with indirect findings.

Case	Age (Years)	Sex	Symptom	Opportunity	Part of the Main Lesion	Type of Echoendoscopes	Background(Early Chronic Pancreatitis/ Chronic Pancreatitis)	Indirect Findings					Further Examination (Single Cytology, SPACE, EUS-FNA, Follow Up)	Final Diagnosis	Undetected Indirect Findings by Previous CT/MRI
								Part	Caliber Change	Retention Cyst	Branch Duct Dilatation	Slightly Low Echoic Lesion (mm)			
1	70	Male	No	PC	Body	Convex	No/No	Body	+	−	−	10	SPACE	PC (pT1b)	None
2	68	Female	Yes	BD-IPMN	Tail	Radial	No/No	Tail	+	−	+	3	Single cytology	IPMN and retention cyst	Slightly low echoic lesion
3	69	Female	No	PC	Body	Radial	No/No	Body	+	−	−	11.2	EUS-FNA	PC (pT2)	None
4	54	Male	No	PC	Neck	Radial	No/No	Neck	+	−	−	10.7	EUS-FNA	PC (pT1c)	None
5	59	Female	No	BD-IPMN	Body	Radial	No/No	Tail	−	−	+	13.6	Follow up	IPMN	Slightly low echoic lesion
6	85	Male	No	BD-IPMN	Body	Radial	No/No	Body	+	−	+	−	Follow up	IPMN	-
7	83	Female	No	BD-IPMN	Head	Radial	No/No	Tail	+	−	−	12	SPACE	IPMN	Slightly low echoic lesion
8	78	Male	No	BD-IPMN	Head	Radial	No/Yes	Body	+	−	+	3.9	SPACE	IPMN	Caliber change, Slightly low echoic lesion
9	88	Male	No	BD-IPMN+IPNB	Tail	Radial	No/No	Tail	+	−	−	7.5	Single cytology	IPMN	Slightly low echoic lesion
10	51	Male	No	Pancreatic pseudocyst	Body	Radial	No/Yes	Body	+	−	−	10.7	SPACE	Pancreatic pseudocyst	Slightly low echoic lesion
11	59	Male	No	BD-IPMN	Neck	Convex	No/No	Neck	−	−	+	7.5	Follow up	IPMN	Slightly low echoic lesion
12	86	Female	No	Mixed-IPMN	Neck	Radial	Yes/No	Neck	+	−	+	−	SPACE	IPMN	None
13	75	Female	No	BD-IPMN	Head	Radial	No/No	Tail	+	−	+	4	Follow up	IPMN	Caliber change, Branch duct dilatation, Slightly low echoic lesion
14	52	Male	No	BD-IPMN	Tail	Radial	Yes/No	Tail	−	+	−	8	SPACE	IPMN	Slightly low echoic lesion
15	77	Male	Yes	Mixed-IPMN	Body	Convex	No/No	Body	+	+	−	−	Single cytology	IPMN	None
16	54	Male	No	Pancreatitis	Head	Radial	No/No	Body	+	−	−	4.6	SPACE	No remarkable findings	Caliber change, Slightly low echoic lesion
17	72	Female	No	MD-IPMN	Body	Radial	No/No	Body	+	−	−	4	SPACE	IPMA	Slightly low echoic lesion
18	67	Male	No	Mixed-IPMN	Tail	Radial	No/No	Body	+	−	−	9.1	Surgery	IPMC (pTis)	Slightly low echoic lesion
19	71	Male	No	MD-IPMN	Head	Radial	No/No	Head	+	−	+	−	Single cytology	IPMN	None
20	52	Male	Yes	PC	Tail	Radial	No/No	Tail	+	−	+	6.7	SPACE	PC (pT3)	Slightly low echoic lesion

PC: pancreatic cancer, BD- IPMN: branch duct intraductal mucinous neoplasm, MD-IPMN: main duct intraductal mucinous neoplasm, IPMB: intraductal papillary neoplasm of the bile duct, IPMA: intraductal mucinous adenoma, IPMC: intraductal mucinous carcinoma, SPACE: serial pancreatic juice aspiration cytological examination, EUS-FNA: endoscopic ultrasonography-fine needle aspiration biopsy, +: present, −: absent.

## Data Availability

The data presented in this study are available on request from the corresponding author. The data are not publicly available due to privacy issues.
